# Malocclusions in primary teeth and quality of life: perception of preschoolers and their parents/guardians

**DOI:** 10.1590/1807-3107bor-2026.vol40.004

**Published:** 2026-03-06

**Authors:** Laerte José da Silva COQUEIRO, Giovanna Vytoria Marinho SILVESTRE, Cristiane Baccin BENDO, Neusa Barros DANTAS, Francisca Aline da Silva MATIAS, Cacilda Castelo Branco LIMA, Marcoeli Silva MOURA, Lúcia de Fátima Almeida de Deus MOURA, Marina de Deus Moura de LIMA

**Affiliations:** (a) Universidade Federal do Piauí – UFPI, School of Dentistry, Department of Pathology and Clinical Dentistry, Teresina, PI, Brazil.; (b) Universidade Federal de Minas Gerais – UFMG, School of Dentistry, Department of Pediatric Dentistry and Orthodontics, Belo Horizonte, MG, Brazil.; (c) Centro Universitário Facid Wyden, School of Dentistry, Department of Dentistry Teresina, Brazil.

**Keywords:** Malocclusion, Quality of Life, Child,Preschool, Oral Health

## Abstract

Malocclusions in primary teeth affect chewing, speech, and aesthetics. Few studies have assessed their impact on preschoolers’ self-reported quality of life. This study aims to determine the association between malocclusions in primary teeth and preschoolers’ quality of life based on reports from children and their parents/guardians. A cross-sectional study was conducted Using a stratified, simple, randomly probabilistic sample. Based on sample size estimation, the study included 566 five-year-old preschoolers and their parents/guardians from Teresina, Brazil. Pediatric Quality of Life Inventory^TM^ questionnaire and sociodemographic forms were applied, and clinical examination was performed. The dmft index, modified developmental defects of enamel, and Foster & Hamilton criteria were used for diagnosis. Descriptive analysis and Poisson regression were performed (p < 0.05; 95% CI). Prevalence of malocclusion was 51.2%. Parents/guardians reported edge-to-edge overjet negatively affected physical capacity (RR = 1.11; 95%CI: 1.02–1.20) and social aspects (RR = 1.11; 95%CI: 1.03–1.19). According to children’s self-reports, posterior crossbite affected the total score (RR = 1.43; 95%CI: 1.03–1.97) and oral health (RR = 1.08; 95%CI: 1 .04–1.13), whereas reduced overbite was linked to negative effects on the emotional aspect (RR = 1.10; 95%CI: 1.01–1.18) and oral health (RR = 1.08; 95%CI: 1.01–1.16). Malocclusions have a negative impact on the quality of life of preschoolers, as reported by parents and children.

## Introduction

Malocclusions are disorders of the dentomaxillofacial complex, with a global prevalence of 54.8% in the primary dentition.^1^ Malocclusion can impair masticatory function, phonation, and aesthetics, in addition to causing psychological disorders in affected patients.^2,3^ In this context, malocclusions can negatively impact the oral health-related quality of life (OHRQoL) of children.^4-6^

OHRQoL is a patient-reported outcome that allows evaluating the extent of functional impairment associated with pain, discomfort, social disability, and psychological well-being caused by an oral condition in daily activities.^6-,9^ OHRQoL questionnaires assess functional, emotional, contextual, and social aspects of children’s lives and those of their families.^10^

Studies on the impact of malocclusion on the OHRQoL of preschoolers have reported conflicting results,^11,12^ which may be associated with the different OHRQoL questionnaires used, such as the “Early Childhood Oral Health Impact Scale” (ECOHIS) and the “Dental Discomfort Questionnaire” (DDQ).^13,14^ These instruments are completed only by guardians, based on the assumption that preschool children do not have the maturity to assess their own quality of life.^13^ Children’s self-reports are, however, of fundamental importance, given that they are able to express their perceptions of OHRQoL even at an early age.^15,16^

Another aspect to highlight is that the questionnaires used in most studies with preschoolers did not evaluate aspects related to children’s general health.^11,5^ The “Pediatric Quality of Life Inventory” (PedsQL) was developed for children aged 2 to 18 years and enables a global analysis of HRQoL, incorporating oral health and general health scales and accounting for the responses of children and their parents.^17^.

Knowing the impact of malocclusion on OHRQoL is important for developing public health policies and allocating public resources.^15^ The present study innovates by using the PedsQL questionnaire to assess OHRQoL in preschoolers. Therefore, this study aimed to evaluate the association between malocclusions in primary teeth and the quality of life of preschoolers, based on reports from children and their parents/guardians.

## Methods

### Study design and ethical aspects

This cross-sectional observational study was approved by the Research Ethics Committee of the Federal University of Piauí (CAAE process number: 817193). This study was conducted in accordance with the “Strengthening the reporting of observational studies in epidemiology” (STROBE) statement.

### Study location

This study was conducted in the city of Teresina, the state capital of Piauí, Brazil. Teresina has a total area of 1,391.293 km^2^, an estimated population of 814,230 inhabitants, a Human Development Index (HDI) of 0.751, and an oral health coverage of 92%. In 2014, Teresina had a total of 259 preschool institutions, comprising 153 public and 106 private schools, and the school enrollment rate was 97.8%.

### Population and sample

The study population consisted of five-year-old preschoolers enrolled in public and private institutions in the city of Teresina, Piauí, Brazil. A stratified, simple, and randomly probabilistic sample was used, and sample size was calculated using the equation $n=z^{2}\cdot p\cdot (1-p)/e^{2}$ , where “z” is the quantile of the normal distribution (for a 95% confidence interval, z = 1.96), “p” is the estimated variation for malocclusion (50%), and “e” is the margin of error (5%). Cochran’s correction for finite populations was then applied, n=n0/(1+n0/N) , where n0 is the initial sample size and N is the population size (7,792 preschoolers). An ideal sample of 365 children was initially determined for the study. Given the multi-stage sampling approach, the design effect was corrected by a factor of 1.5(365×1.5=547) . To minimize possible losses, the sample size was increased by 10%, resulting in an ideal sample of 602(547+55=602) preschoolers.

The sample was proportionally stratified according to the type of preschool (public or private) and the city’s districts (north, south, southeast, and east). In 2014, approximately 60% of the preschools were public and 40% were private. Based on this distribution, five preschools were randomly selected from each district — three public and two private — resulting in a total of 25 preschools. Classes with 5-year-old children were identified within each institution, and attendance lists were obtained. Based on the attendance lists, an average of 26 children per public school and 24 children per private school were randomly selected until the target sample size of 602 children was achieved.

### Inclusion and exclusion criteria

Preschoolers who were aged five years on the date of the clinical examination and who had complete primary teeth were included. Individuals with autism spectrum disorder and children who were uncooperative during the clinical examination were excluded from the research.

### Calibration

The calibration exercise was carried out in two phases. The theoretical-practical phase involved discussion of the diagnostic criteria for malocclusion, developmental defects of enamel (DDE), and dental caries, according to the Foster & Hamilton criteria, modified DDE index, and dmft index, respectively. At this stage, photographs of teeth with/without malocclusion, developmental defects of enamel, and dental caries were analyzed. The theoretical-practical phase of calibration was led by a pediatric dentistry and orthodontics specialist with experience in epidemiological studies. The clinical phase was conducted during the pilot study, in which intra-examiner agreement was determined by clinical evaluation of the children at two time points with a minimum interval of 15 days between the examinations. Intra-examiner agreement, assessed using Cohen’s kappa coefficient, was 0.92 for malocclusion, 1.00 for DDE, and 0.96 for dental caries.

### Pilot study

A pilot study was carried out before participant selection with the objective of adapting the research methodology (for approaching children and guardians) and evaluating the reliability and validity of the quality-of-life questionnaire for this study. The study was carried out with 60 children (10% of the sample) at three day care centers (two public and one private). These children were not included in the final research sample. The results of the pilot study demonstrated that the proposed methodology was appropriate and required no adjustments.

### Data collection and analysis

After the pilot study, data were collected between October and December 2014 using three approaches: a) a printed sociodemographic questionnaire for parents or guardians; b) a printed validated quality-of-life instrument (*Pediatric Quality of Life Inventory* - PedsQL); and c) a clinical dental examination, performed at the schools by trained and calibrated examiners under standardized conditions. The printed questionnaires were sent home with the children for completion by parents/guardians and later returned in sealed envelopes.

Five preschools were randomly drawn by the regional superintendencies (north, south, east, and southeast) of Teresina, and stratified according to type of preschool (public or private). In each selected preschool, the attendance lists of the identified classes of 5-year-olds were compiled and organized alphabetically. Each child was assigned a unique sequential number, and participants were randomly selected.

### Sociodemographic characteristics

Sociodemographic characteristics were collected using a self-administered questionnaire sent home to guardians The questionnaire contained questions about the child’s sex, family income (in minimum wages), mother’s and father’s education (in years of formal study), type of preschool (public or private), and history of dental trauma. Family income was categorized based on the 2014 Brazilian monthly minimum wage (approximately US$246.40). Level of education of guardians was dichotomized using a cut-off point of eight years, which corresponds to the level of primary education in Brazil.

### Quality of Life Questionnaire (PedsQL)

The PedsQL^TM^Oral Health Scale and PedsQL^TM^4.0 Generic Core Scales (Brazilian Portuguese version)^21,22^ questionnaires were administered to children through interviews conducted at school before the examination. The questionnaires intended for parents were sent home with the children and self-administered. The PedsQL^TM^4.0 Generic Core Scales questionnaire consists of 23 items organized into four domains: physical capacity (eight items), emotional aspect (five items), social aspect (five items) and school activity (five items), whereas the PedsQL ^TM^Oral Health Scale questionnaire consists of five items. Responses on the guardians’ questionnaire were recorded using a five-point scale (100 = never a problem; 75 = almost never a problem; 50 = sometimes a problem; 25 = often a problem; 0 = almost always a problem). The following simplified facial hedonic scale was used to facilitate the application of the questionnaire to children: 100 = never a problem; 50 = sometimes a problem; 0 = almost always a problem. Quality of life was measured by the average score obtained for each domain and for the total questionnaire. Higher scores indicated better quality of life.^22^

### Dental examination

A dental examination was performed to assess dental caries , presence of malocclusions, and DDE. The assessment was carried out by a single previously trained and calibrated examiner in a classroom within the educational institution where the child was enrolled. Before the examination, the children’s teeth were cleaned using a toothbrush and toothpaste. The children were examined under artificial lighting (Pelicano® table lamp – Startec with 127V, São Paulo, Brazil) in a simplified position, with the child’s head resting on the examiner’s legs. Sterile gauze compresses were used to dry the teeth, and the examinations were performed with the aid of a flat mouth mirror (Golgran®, São Paulo, Brazil), exploratory probe # 5 (Golgran®, São Paulo, Brazil), and a probe recommended by the World Health Organization (WHO) (Trinity®, São Paulo, Brazil).

Dental caries was diagnosed using the dmft index, as recommended by WHO.^23^ The diagnosis of malocclusion was based on Foster and Hamilton^24^ and Grabowski et al.^25^ criteria for the primary dentition. Children were considered to have malocclusion if they presented at least one of the following conditions: a class II or class III canine relationship; reduced (≤ 2 mm), deep (≥ 2 mm), or open overbite; increased overjet (≥ 2 mm); anterior crossbite (≤ 2 mm), or edge-to-edge and posterior crossbite. The modified DDE index recommended by the International Dental Federation (FDI) (1992) was used to diagnose enamel defects. Caries experience and the presence of DDE were evaluated as possible confounding variables in the assessment of quality of life.

### Statistical analysis

The data were analyzed using the Statistical Package for the Social Sciences (SPSS® for Windows, version 20.0, SPSS Inc., Chicago, USA). Cronbach’s alpha test was used to assess the reliability of children’s responses to the questionnaire, while and test-retest validity was evaluated using the intraclass correlation coefficient. A descriptive analysis was conducted using absolute and relative frequencies, mean, standard deviation and median. The data distribution hypothesis for the general result and the PedsQL domains followed the Poisson distribution.

Poisson regression with robust variance was performed to determine the joint effect on PedsQL domain scores and total scores for malocclusion, DDE, dental caries, history of dental trauma, and sociodemographic characteristics. Bivariate analysis was used to compare two distributions, evaluate mean differences in in the quality-of-life questionnaire scores and independent variables, and select variables with a p-value ≤ 0.20 to be tested in the regression model. The explanatory variables selected from the guardians’ questionnaire were tested in a multivariate model adjusted for dental caries. Given the similarity in the children’s questionnaire mean scores for dental caries, the model was adjusted for the guardians’ level of education. The results were expressed as rate ratio (RR) and 95% confidence interval (95% CI). Associations with a p < 0.05 were kept in the model. A significance level of α = 5% was considered for all analyses.

## Results

A total of 566 children (94%) participated in this study (Table 1). Loss to follow-up amounted to 6%. On the day of the examination, 36 children were excluded from the study: 17 (2.8%) were absent, four (0.7%) had been diagnosed with autism spectrum disorder, and 15 (2.5%) were aged > 5 years.

**Table 1:** Socioeconomic and sociodemographic characteristics and dental clinical conditions of the sample (n = 566).

Peds domains	Physical functioning	Emotional functioning	Social functioning	School functioning	Total generic core scale	Oral health scale
	RRadj (95%CI)	RRadj (95%CI)	RRadj (95%CI)	RRadj (95%CI)	RRadj (95%CI)	RRadj (95%CI)
Sex						
Male	–	–	1.04 (101–1.08)	1.05 (1.01–1.10)	1.15 (095–1.39)	1.02 (0.99–1.05)
Female			1	1	1	1
Family income (MW)						
≤ 2	1.08 (1.01–1.16)	–	1.07 (1.01–1.12)	1.08 (0.99–1.17)	1.07 (0.85–1.24)	1.02 (0.95–1.07)
> 2	1		1	1	1	1
Mother’s education (years of study)						
≤ 8	–	–	1.01 (0.95–1.07)	1.02 (0.96–1.09)	1.22 (1.02–1.45)	1.02 (0.95–1.07)
> 8			1	1	1	1
Father’s education (years of study)						
≤ 8	1.02 (0.98–1.06)	1.03 (0.98–1.07)	1.03 (0.98–1.08)	1.02 (0.96–1.07)	1.07 (0.83–1.26)	1.04 (1.01–1.08)
> 8	1	1	1	1	1	1
Type of school						
Public	1.07 (1.01–1.15)	–	1.01 (0.95–1.08)	1.02 (0.92–1.13)	1.10 (0.90–1.34)	1.01 (0.95–1.07)
Private	1		1	1	1	1
History of dental trauma						
Yes	1.07 (1.02–1.12)	1.07 (1.01–1.13)	–	–	1.12 (0.95–1.32)	1.10 (1.06–1.15)
No	1	1			1	1
Malocclusion						
Yes	1.02 (0.98–1.06)	–	–	–	–	–
No	1					
Dmft						
> 0	1.04 (1.01–108)	1.06 (1.01–1.11)	1.03 (0.99–1.07)	1.05 (1.01–1.10)	1.17 (1.01–1.37)	1.06 (1.04–1.09)
= 0	1	1	1	1	1	1
Overjet						
Normal	1		1			
Anterior crossbite	0.96 (0.86–1.03)	–	0.92 (0.83–102)	–	–	–
Increased	1.02 (0.96–1.07)		1.01 (0.95–1.07)			
Edge-to-edge	1.11 (1.02–1.20)		1.11 (1.03–1.19)			

Rradj: adjusted rate ratio; 95%CI: 95% confidence interval; MW: minimum wages; DDE: developmental defects of enamel; dmft: decayed, missing, and filled teeth.

Quality-of-life perceptions were assessed (Cronbach’s alpha = 0.724; test-retest = 0.726, 95% CI = 0.541–0.836).

The sociodemographic and clinical characteristics of the study are described in Table 1. Malocclusion was diagnosed in 290 children (51.2%). Furthermore, approximately 50% of the children had caries experience and 33.7% had DDE (Table 1).

Class II canine relationship was the most common type of malocclusion (21.3%), followed by increased overjet (15.2%) and reduced overbite (14%). Posterior crossbite was present in 7.1% of the preschool children evaluated.

Mean, standard deviation, median, and bivariate analysis of sociodemographic and clinical characteristics and the quality-of-life scores for each domain and the total score are described in Tables 2, 3,4, and 5. In the unadjusted analysis, no association was found between the presence of malocclusion in children and poorer quality of life. Upon the assessment of the occlusion criteria, an association was observed between posterior crossbite and worse overall quality-of-life scores (p = 0.039), and reduced overbite was associated with lower scores in the oral health domain (p = 0.019), according to children’s self-reports. Based on the guardians’ reports, edge-to-edge overjet had a negative impact on quality of life in the social domain (p = 0.041).

**Table 2:** Bivariate analysis of associations between the overall score and PedsQL domains and independent variables based on children’s reports

Peds domains	Physical functioning		Emotional functioning		Social functioning		School functioning		Total Generic Core Scale		Oral Health Scale	
	µ (SD)	Med	µ (SD)	Med	µ (SD)	Med	µ (SD)	Med	µ (SD)	Med	µ (SD)	Med
Sex												
Male	70.93 (17.91)	68.77	67.17 (24.18)	70.00	70.13 (21.13)	70.00	70.49 (21.65)	70.00	69.84 (15.62)	69.56	74.15 (23.31)	80.00
Female	66.01 (17.81)	62.50	59.66 (25.83)	60.00	72.18 (20.84)	70.00	71.73 (20.92)	70.00	67.22 (15.86)	67.39	72.49 (26.00)	80.00
p-value*	0.002		<0.001		0.244		0.489		0.061		0.426	
Family income (MW)												
≤ 2	66.65 (18.23)	62.50	61.25 (25.76)	60.00	70.34 (21.14)	70.00	69.84 (22.67)	70.00	66.97 (16.34)	67.39	70.47 (25.62)	80.00
> 2	72.72 (16.89)	75.00	68.64 (23.39)	70.00	72.66 (20.69)	70.00	73.64 (17.93)	80.00	72.02 (13.97)	71.74	79.40 (21.17)	80.00
p-value*	<0.001		0.001		0.211		0.030		<0.001		<0.001	
Mother’s education (years of study)												
≤ 8	63.87 (17.72)	62.50	58.86 (26.45)	60.00	66.93 (22.16)	60.00	65.66 (21.47)	60.00	63.84 (16.62)	60.87	64.46 (26.12)	60.00
> 8	70.34 (17.83)	68.75	65.38 (24.58)	60.00	72.59 (20.39)	70.00	73.02 (20.92)	80.00	70.34 (15.11)	69.56	76.58 (23.24)	80.00
p-value*	<0.001		0.010		0.007		<0.001		<0.001		<0.001	
Father’s education (years of study)												
≤ 8	65.21 (18.41)	62.50	61.72 (25.47)	60.00	68.57 (22.80)	70.00	68.91 (23.14)	70.00	65.98 (16.70)	65.21	67.43 (25.64)	70.00
> 8	70.54 (17.53)	68.75	64.74 (25.06)	60.00	72.50 (19.82)	70.00	72.28 (20.13)	80.00	70.08 (15.06)	69.56	76.69 (23.39)	80.00
p-value*	<0.001		0.177		0.041		0.084		0.003		<0.001	
Type of school												
Public	66.79 (18.27)	62.50	61.63 (25.99)	60.00	70.18 (21.09)	70.00	69.94 (22.47)	70.00	67.09 (16.32)	67.39	70.16 (25.71)	70.00
Private	72.38 (16.93)	75.00	67.79 (23.11)	70.00	72.95 (20.75)	70.00	73.38 (18.53)	80.00	71.72 (14.14)	71.74	79.94 (20.73)	80.00
p-value*	<0.001		0.004		0.134		0.052		0.001		<0.001	
History of dental trauma												
Yes	67.04 (17.54)	62.50	63.36 (26.99)	60.00	69.89 (22.62)	70.00	69.79 (21.88)	70.00	67.46 (16.51)	67.39	70.84 (21.66)	70.00
No	68.94 (18.11)	68.75	63.71 (24.89)	60.00	71.33 (20.68)	70.00	71.33 (21.20)	70.00	68.85 (15.63)	69.56	73.88 (25.14)	80.00
p-value*	0.175		0.908		0.566		0.528		0.287		0.228	
Malocclusion												
Yes	68.58 (18.19)	68.75	63.55 (25.63)	60.00	71.24 (20.92)	70.00	71.21 (21.54)	80.00	68.63 (16.15)	69.56	72.24 (24.28)	80.00
No	68.68 (17.87)	68.75	63.79 (24.85)	60.00	70.94 (21.13)	70.00	70.94 (21.09)	70.00	68.59 (15.41)	69.56	74.56 (24.92)	80.00
p-value*	0.239		0.919		0.865		0.882		0.348		0.260	
Dmft												
> 0	67.45 (18.97)	68.75	63.48 (25.90)	60.00	71.62 (22.13)	70.00	71.16 (21.93)	70.00	68.30 (17.02)	69.56	68.76 (26.53)	70.00
= 0	69.81 (16.96)	68.75	63.83 (24.58)	60.00	70.57 (19.83)	70.00	70.99 (20.69)	70.00	68.93 (14.44)	69.56	78.01 (21.56)	80.00
p-value*	0.103		0.871		0.550		0.925		0.664		<0.001	
DDE												
Yes	67.70 (17.85)	68.75	62.56 (24.71)	60.00	70.52 (22.66)	70.00	71.72 (20.61)	70.00	68.07 (15.71)	69.56	71.52 (25.71)	80.00
No	69.10 (18.11)	68.75	64.21 (25.51)	60.00	71.39 (20.14)	70.00	70.75 (21.67)	70.00	68.89 (15.82)	69.56	76.32 (23.99)	80.00
p-value*	0.282		0.459		0.657		0.597		0.568		0.001	
Total	68.63 (18.02)	68.75	63.66 (25.23)	60.00	71.09 (21.01)	70.00	71.08 (21.30)	70.00	68.62 (15.78)	69.56	73.37 (24.60)	80.00

µ: average; SD: standard deviation; Med: median; MW: minimum wages; DDE: developmental defects of enamel; dmft: decayed, missing, and filled teeth.

*Poisson test.

**Table 3:** Bivariate analysis of associations between the overall score and PedsQL domains and independent variables based on parents’ or guardians’ reports.

Peds domains	Physical functioning		Emotional functioning		Social functioning		School functioning		Total Generic Core Scale		Oral Health Scale	
	µ (SD)	Med	µ (SD)	Med	µ (SD)	Med	µ (SD)	Med	µ (SD)	Med	µ (SD)	Med
Sex												
Male	80.25 (19.34)	84.37	71.19 (18.84)	70.00	79.60 (19.86)	85.00	68.30 (20.73)	70.00	75.54 (15.65)	79.34	88.25 (15.73)	95.00
Female	81.91 (18.71)	87.50	70.49 (17.60)	70.00	82.64 (19.09)	90.00	71.64 (19.83)	75.00	77.35 (15.12)	80.43	90.35 (14.24)	95.00
p-value*	0.308		0.645		0.063		0.050		0.186		0.084	
Family income (MW)												
≤ 2	79.31 (20.02)	84.37	70.68 (19.32)	70.00	78.48 (21.12)	85.00	67.16 (21.04)	70.00	74.61 (16.40)	77.17	87.49 (15.61)	90.00
> 2	84.59 (16.34)	90.62	71.25 (15.87)	70.00	86.30 (14.44)	90.00	75.49 (17.64)	80.00	80.08 (12.40)	81.52	92.88 (11.18)	100.00
p-value*	0.007		0.709		<0.001		<0.001		0.001		<0.001	
Mother’s education (years of study)												
≤ 8	79.91 (20.02)	87.50	69.76 (20.09)	70.00	78.80 (22.05)	85.00	66.46 (21.93)	70.00	74.54 (16.69)	77.17	85.50 (18.04)	90.00
> 8	81.43 (18.69)	87.50	71.26 (17.56)	70.00	81.82 (18.52)	85.00	71.09 (19.65)	70.00	77.06 (14.90)	79.34	90.59 (12.79)	95.00
p-value*	0.482		0.421		0.137		0.025		0.002		0.002	
Father’s education (years of study)												
≤ 8	79.69 (19.18)	84.37	69.06 (19.71)	70.00	78.22 (20.54)	80.00	68.00 (21.08)	70.00	74.52 (15.91)	76.08	85.12 (18.07)	90.00
> 8	81.77 (18.96)	87.50	71.87 (17.34)	70.00	82.59 (18.81)	90.00	70.91 (19.91)	70.00	77.43 (15.06)	80.43	91.54 (11.50)	95.00
p-value*	0.157		0.092		0.013		0.111		0.029		<0.001	
Type of school												
Public	79.30 (20.21)	84.37	70.43 (19.49)	70.00	78.72 (21.16)	85.00	67.35 (21.15)	70.00	74.65 (16.55)	77.17	87.39 (15.93)	90.00
Private	84.56 (15.88)	90.62	71.74 (15.45)	70.00	85.72 (14.71)	90.00	75.00 (17.61)	75.00	79.94 (12.07)	81.52	93.01 (10.16)	95.00
p-value*	0.015		0.383		<0.001		<0.001		0.001		<0.001	
History of dental trauma												
Yes	77.89 (20.37)	84.37	66.52 (19.28)	70.00	80.21 (20.29)	85.00	68.31 (20.21)	70.00	73.84 (16.38)	77.17	81.21 (16.23)	85.00
No	81.66 (18.73)	87.50	71.74 (17.94)	70.00	81.19 (19.41)	85.00	70.18 (20.40)	70.00	76.90 (15.18)	79.34	90.86 (13.62)	95.00
p-value*	0.054		0.017		0.666		0.415		0.109		<0.001	
Malocclusion												
Yes	80.09 (18.66)	84.37	70.38 (18.07)	70.00	80.67 (19.62)	85.00	69.20 (19.75)	70.00	75.74 (15.33)	79.34	89.32 (13.68)	95.00
No	82.00 (19.43)	89.06	71.37 (18.48)	70.00	81.39 (19.48)	85.00	70.56 (21.00)	70.00	77.07 (15.51)	79.34	89.14 (15.39)	95.00
p-value*	0.105		0.516		0.660		0.429		0.304		0.884	
dmft												
> 0	79.50 (19.29)	84.37	68.48 (19.05)	70.00	78.71 (20.76)	85.00	67.27 (21.26)	70.00	74.27 (15.81)	76.08	85.24 (16.97)	90.00
= 0	82.56 (18.71)	87.50	73.26 (17.12)	75.00	83.35 (17.97)	90.00	72.48 (19.10)	75.00	78.52 (14.73)	80.97	93.26 (10.10)	100.00
p-value*	0.039		0.002		0.005		0.002		0.001		<0.001	
DDE												
Yes	81.47 (18.42)	84.37	71.09 (18.18)	70.00	81.25 (19.39)	85.00	70.75 (20.32)	75.00	76.84 (15.28)	80.43	89.50 (14.93)	95.00
No	80.80 (19.38)	87.50	70.74 (18.32)	70.00	80.90 (19.64)	85.00	69.41 (20.40)	70.00	76.16 (15.50)	79.34	89.10 (14.34)	95.00
p-value*	0.860		0.827		0.839		0.455		0.608		0.762	
Total	81.03 (19.05)	87.50	70.86 (18.32)	70.00	81.02 (19.54)	85.00	69.86 (20.36)	70.00	76.39 (15.42)	79.34	89.24 (14.53)	95.00

µ: average; SD: standard deviation; Med: median; MW: minimum wages; DDE: developmental defects of enamel; dmft: decayed, missing, and filled teeth. *Poisson test.

**Table 4:** Association between general and oral health-related quality of life and occlusion parameters based on children’s reports.

Peds domains	Physical functioning		Emotional functioning		Social functioning		School functioning		Total generic core scale		Oral health scale	
	µ (SD)	Med	µ (SD)	Med	µ (SD)	Med	µ (SD)	Med	µ (SD)	Med	µ (SD)	Med
Canine relationship												
Class I	68.32 (18.24)	68.75	64.22 (24.69)	60.00	71.61 (20.86)	70.00	71.24 (21.51)	70.00	68.78 (15.49)	69.56	74.75 (24.47)	80.00
Class II	69.83 (17.78)	68.75	62.64 (27.04)	60.00	70.08 (20.91)	70.00	70.25 (21.11)	70.00	68.41 (16.68)	69.56	71.07 (24.14)	80.00
Class III	68.18 (17.29)	68.75	62.27 (25.16)	60.00	70.00 (22.18)	70.00	71.66 (22.77)	80.00	68.05 (15.90)	68.48	69.69 (25.83)	70.00
p-value*	0.769		0.781		0.700		0.806		0.941		0.215	
Posterior crossbite												
Yes	63.59 (15.75)	62.50	56.75 (25.75)	50.00	69.75 (21.89)	70.00	65.50 (25.01)	70.00	63.85 (15.07)	63.04	67.75 (25.57)	70.00
No	69.01 (18.13)	68.75	64.18 (25.14)	60.00	71.19 (20.95)	70.00	71.50 (20.96)	70.00	68.97 (15.78)	69.56	73.80 (24.50)	80.00
p-value*	0.070		0.086		0.704		0.168		**0.039**		0.125	
Overbite												
Normal	69.12 (17.77)	68.75	65.00 (24.55)	60.00	71.27 (21.18)	70.00	71.30 (21.22)	70.00	69.17 (15.39)	69.56	74.06 (24.24)^a^	80.00
Reduced	65.58 (18.74)	62.50	57.59 (24.50)	50.00	71.64 (20.15)	80.00	68.99 (21.46)	70.00	65.90 (16.19)	69.56	65.82 (26.58)^b^	70.00
Open	64.11 (16.92)	62.50	62.58 (24.49)	60.00	69.35 (24.07)	70.00	73.22 (23.29)	80.00	66.90 (16.81)	67.39	73.22 (23.15)^a.b^	80.00
Deep	71.26 (18.57)	75.00	63.61 (29.13)	60.00	70.28 (19.93)	70.00	71.25 (20.96)	70.00	69.38 (16.82)	69.56	78.06 (23.59)^a^	80.00
p-value*	0.127		0.100		0.950		0.715		0.372		**0.019**	
Overjet												
Normal	69.11 (17.88)	68.75	63.93 (24.72)	60.00	70.57 (20.73)	70.00	70.85 (21.25)	70.00	68.68 (15.53)	69.56	74.41 (24.42)	80.00
Increased	68.53 (18.55)	62.50	64.18 (28.05)	70.00	72.56 (21.86)	80.00	70.58 (22.08)	70.00	68.90 (16.97)	69.56	71.39 (22.86)	70.00
Edge-to-edge	61.50 (16.50)	62.50	59.60 (23.88)	60.00	72.40 (23.32)	80.00	76.00 (20.61)	80.00	66.61 (14.09)	69.56	70.40 (27.46)	70.00
Anterior crossbite	67.50 (19.82)	62.50	60.50 (26.45)	60.00	74.50 (21.14)	80.00	72.00 (20.67)	70.00	68.48 (18.53)	66.30	63.00 (30.28)	65.00
p-value*	0.343		0.725		0.636		0.677		0.965		0.207	
Total	68.63 (18.02)	68.75	63.66 (25.23)	60.00	71.09 (21.01)	70.00	71.08 (21.30)	70.00	68.62 (15.78)	69.56	73.37 (24.60)	80.00

µ: average; SD: standard deviation; Med: median. * Poisson test. Different letters indicate a p < 0.05.

**Table 5:** Association between general and oral health-related quality of life and occlusion parameters based on parents’ or guardians’ reports

Peds domains	Physical functioning		Emotional functioning		Social functioning		School functioning		Total generic core scale		Oral health scale	
	µ (SD)	Med	µ (SD)	Med	µ (SD)	Med	µ (SD)	Med	µ (SD)	Med	µ (SD)	Med
Canine relationship												
Class I	81.55 (19.02)	87.50	71.26 (17.80)	70.00	81.29 (19.32)	85.00	70.35 (20.40)	70.00	76.82 (15.14)	79.34	89.47 (14.35)	95.00
Class II	80.55 (19.32)	84.37	71.23 (19.11)	70.00	80.12 (20.86)	90.00	69.09 (20.50)	70.00	75.94 (16.42)	79.34	88.84 (15.62)	95.00
Class III	78.88 (18.79)	84.37	67.87 (19.31)	65.00	81.13 (18.58)	82.50	68.48 (20.07)	70.00	74.72 (15.19)	78.80	88.63 (13.63)	95.00
p-value*	0.357		0.298		0.886		0.749		0.558		0.788	
Posterior crossbite												
Yes	81.64 (14.88)	84.37	69.00 (20.88)	70.00	81.12 (16.73)	80.00	70.75 (21.22)	75.00	76.41 (13.49)	79.34	87.50 (15.06)	92.50
No	80.98 (19.34)	87.50	71.00 (18.06)	70.00	81.01 (19.75)	85.00	69.80 (20.31)	70.00	76.39 (15.57)	79.34	89.37 (14.49)	95.00
p-value*	0.620		0.564		0.767		0.759		0.797		0.475	
Overbite												
Normal	81.73 (19.23)	87.50	72.01 (17.72)	70.00	81.53 (19.11)	85.00	70.26 (20.47)	70.00	77.08 (15.02)	79.34	88.71 (15.15)	95.00
Reduced	78.40 (19.77)	81.25	68.86 (20.89)	70.00	80.44 (21.85)	90.00	66.20 (19.74)	65.00	74.12 (16.77)	78.26	90.19 (13.50)	95.00
Open	78.63 (16.96)	78.12	66.61 (15.51)	65.00	78.87 (18.19)	80.00	73.70 (19.57)	75.00	75.00 (14.06)	79.34	90.16 (11.29)	95.00
Deep	81.20 (18.09)	84.37	68.75 (18.79)	70.00	79.86 (19.98)	87.50	70.13 (20.67)	70.00	75.80 (16.49)	79.34	90.62 (13.50)	95.00
p-value*	0.314		0.170		0.679		0.251		0.518		0.608	
Overjet												
Normal	81.38 (19.36)	87.50	71.43 (17.84)	70.00	81.24 (19.00)^a^	85.00	70.20 (20.28)	70.00	76.76 (15.09)	79.34	89.23 (14.53)	95.00
Increased	81.14 (17.34)	84.37	69.30 (19.73)	70.00	80.46 (22.64)^a^	90.00	68.54 (19.94)	70.00	75.68 (16.84)	79.34	88.95 (14.53)	95.00
Edge-to-edge	74.00 (18.23)	75.00	64.60 (18.53)	70.00	73.20 (19.41)^b^	75.00	71.00 (20.81)	70.00	71.13 (15.15)	72.82	91.20 (9.81)	90.00
Anterior crossbite	81.56 (19.84)	84.37	73.00 (19.82)	70.00	88.50 (14.05)^a^	95.00	66.75 (24.34)	75.00	77.99 (16.26)	82.06	88.25 (17.71)	95.00
p-value*	0.154		0.313		**0.041**		0.877		0.300		0.975	
Total	81.02 (19.05)	87.50	70.86 (18.26)	70.00	81.02 (19.54)	85.00	69.86 (20.36)	70.00	76.39 (15.42)	79.34	89.24 (14.53)	95.00

µ: average; SD: standard deviation; Med: median. * Poisson test. Different letters indicate a p < 0.05.

Multivariate analyses (Tables 6 and 7) revealed that children with posterior crossbite exhibited worse quality of life, as reflected in the total score (RR = 1.43; 95% CI = 1.03–1.97) and in the oral health (RR = 1.10; 95%CI: 1.04–1.13) than children without the condition. Children with reduced overbite were more likely to have a negative impact on quality of life in the emotional domain (RR = 1.10; 95%CI: 1.01–1.18) and oral health domain (RR = 1.08; 95%CI: 1.01–1.16) (Table 6). The presence of edge-to-edge overjet had a negative impact on the physical capacity (RR = 1.11; 95%CI: 1.02–1.20) and social (RR = 1.11; 95%CI: 1.03–1.19) domains, as reported by guardians (Table 7).

**Table 6:** Multivariate Poisson regression model for general score and PedsQL domains and independent variables based on children’s reports.

Peds domains	Physical functioning	Emotional functioning	Social functioning	School functioning	Total generic core scale	Oral health scale
	RRadj (95%CI)	RRadj (95%CI)	RRadj (95%CI)	RRadj (95%CI)	RRadj (95%CI)	RRadj (95%CI)
Sex						
Male	**0.86 (0.80–0.93)**	**0.89 (0.83–0.95)**	**-**	-	0.91 (0.83–1.01)	**-**
Female	1	1			1	
Family income (MW)						
≤ 2	**1.39 (1.18–1.63)**	**1.14 (1.02–1.27)**	-	1.05 (0.95–1.15)	**1.60 (1.18–2.18)**	**1.05 (1.01–1.09)**
> 2	1	1		1	1	1
Mother’s education (years of study)						
≤ 8	1.06 (0.95–1.18)	1.07 (0.98–1.18)	**1.07 (1.01–1.13)**	**1.10 (1.03–1.18)**	**1.11 (1.02–1.21)**	**1.11 (1.02–1.20)**
> 8	1	1	1	1	1	1
Father’s education (years of study)						
≤ 8	1.08 (0.98–1.16)	1.02 (0.94–1.10)	1.03 (0.97–1.09)	1.00 (0.94–1.07)	**1.14 (1.04–1.24)**	1.04 (0.99–1.08)
> 8	1	1	1	1	1	1
Type of school						
Public	**1.33 (1.12–1.57)**	1.05 (0.94–1.17)	1.01 (0.95–1.06)	1.03(0.93–1.14)	**1.66 (1.24–2.21)**	1.04 (0.94–1.13)
Private	1	1	1	1	1	1
History of dental trauma						
Yes	1.06 (0.97–1.15)	-	**-**	-	-	-
No	1					
Dmft						
> 0	**1.11 (1.02–1.21)**	-	**-**	-	-	**1.08 (1.02–1.14)**
= 0	1					1
DDE						
Yes	–	**-**	**-**	-	-	1.02 (0.96–1.08)
No						1
Posterior crossbite						
Yes	1.09 (0.96–1.21)	1.10 (0.96–1.27)	-	1.07 (0.95–1.21)	**1.43 (1.03–1.97)**	**1.08 (1.04–1.13)**
No	1	1		1	1	
Overbite						
Normal	1	1				1
Deep	0.99 (0.90–1.12)	1.01 (0.90–1.12)	-	-	-	0.97 (0.95–1.01)
Reduced	1.09 (0.92–1.29)	**1.10 (1.01–1.18)**				**1.08 (1.01–1.16)**
Open	1.10 (0.99–1.23)	1.01 (0.86–1.13)				1.00 (0.97–1.03)

RRajus: adjusted rate ratio; 95%CI: 95% confidence interval; MW: minimum wages; DDE: developmental defects of enamel; dmft: decayed, missing, and filled teeth. Values in bold indicate a p < 0.05.

**Table 7:** Multivariate Poisson regression model for general score and PedsQL domains and independent variables based on parents’ or guardians’ reports.

Peds domains	Physical functioning	Emotional functioning	Social functioning	School functioning	Total generic core scale	Oral health scale
	RRadj (95%CI)	RRadj (95%CI)	RRadj (95%CI)	RRadj (95%CI)	RRadj (95%CI)	RRadj (95%CI)
Sex						
Male	-	-	1.04 (101–1.08)	1.05 (1.01–1.10)	1.15 (095–1.39)	1.02 (0.99–1.05)
Female			1	1	1	1
Family income (MW)						
≤ 2	1.08 (1.01–1.16)	-	1.07 (1.01–1.12)	1.08 (0.99–1.17)	1.07 (0.85–1.24)	1.02 (0.95–1.07)
> 2	1		1	1	1	1
Mother’s education (years of study)						
≤ 8	-	-	1.01 (0.95–1.07)	1.02 (0.96–1.09)	1.22 (1.02–1.45)	1.02 (0.95–1.07)
> 8			1	1	1	1
Father’s education (years of study)						
≤ 8	1.02 (0.98–1.06)	1.03 (0.98–1.07)	1.03 (0.98–1.08)	1.02 (0.96–1.07)	1.07 (0.83–1.26)	1.04 (1.01–1.08)
> 8	1	1	1	1	1	1
Type of school						
Public	1.07 (1.01–1.15)	-	1.01 (0.95–1.08)	1.02 (0.92–1.13)	1.10 (0.90–1.34)	1.01 (0.95–1.07)
Private	1		1	1	1	1
History of dental trauma						
Yes	1.07 (1.02–1.12)	1.07 (1.01–1.13)	-	-	1.12 (0.95–1.32)	1.10 (1.06–1.15)
No	1	1			1	1
Malocclusion						
Yes	1.02 (0.98–1.06)	-	-	-	-	-
No	1					
Dmft						
> 0	1.04 (1.01–108)	1.06 (1.01–1.11)	1.03 (0.99–1.07)	1.05 (1.01–1.10)	1.17 (1.01–1.37)	1.06 (1.04–1.09)
= 0	1	1	1	1	1	1
Overjet						
Normal	1		1			
Anterior crossbite	0.96 (0.86–1.03)	–	0.92 (0.83–102)	-	-	-
Increased	1.02 (0.96–1.07)		1.01 (0.95–1.07)			
Edge-to-edge	1.11 (1.02–1.20)		1.11 (1.03–1.19)			

RRajus: adjusted rate ratio; 95%CI: 95% confidence interval; MW: minimum wages; DDE: developmental defects of enamel; dmft: decayed, missing, and filled teeth. Values in bold indicate a p < 0.05.

Dental caries had a negative impact on the physical capacity (RR = 1.11; 95% CI = 1.02–1.21) and oral health (RR = 1.08; 95% CI = 1.02–1.14 ) domains, as evidenced in the children’s reports (Table 6). A negative impact was observed for the physical capacity (RR = 1.04; 95%CI: 1.01–1.08), emotional (RR = 1.06; 95%CI: 1.01–1.11), school activity (RR = 1.05; 95%CI: 1.01–1.10), and oral health (RR = 1.06; 95%CI: 1.04–1.09) domains, as well as for the total quality-of-life score (RR = 1.17; 95%CI: 1.01–1.37), as pointed out in the guardians’ reports (Table 7). The presence of DDE was not associated with worse quality of life in the PedsQL domains and total score, based on either the children’s or guardians’ reports (p > 0.05).

## Discussion

To the best of our knowledge, this population-based study is innovative in evaluating the impact of different types of malocclusions on the quality of life of five-year-old children based on their self-reports and on their guardians’ assessments. The present study concluded that malocclusions had a negative impact on the OHRQoL of preschoolers, as reported by both children and their parents.

The findings of this study are at odds with those of most studies in the literature involving this age group, which did not report an association between malocclusions and OHRQoL.^3,12,18^ Some studies, however, investigated only the presence and/or absence of malocclusion, not accounting for the different impact of each type of malocclusion on OHRQoL.^19,20^ The present study assessed the presence and/or presence of malocclusion and the impact on OHRQoL.

In addition, these studies used parent proxy measures in which parents responded for their children.^12,18-20^ Nevertheless, children’s self-perception is important, given that they are capable of evaluating how their appearance interferes with their social relationships and daily activities.^5,15^

Furthermore, parents are not always able to assess the impact of oral conditions on their children’s physical and psychological well-being, as many of these conditions may be overlooked by caregivers.^15^ Malocclusions are asymptomatic, thus hindering their detection by parents or guardians, who are more likely to detect oral conditions that cause pain or discomfort, such as dental caries.^20^ Furthermore, discrepancies in literature findings may also be due to methodological differences, including age group, type of questionnaire, diagnostic criteria, and regional aspects of each population. Such discrepancies preclude comparison between studies.

The PedsQL considers general and specific clinical aspects of oral health and sociodemographic variables.^21^ This questionnaire was selected because it enables comparisons between the perceptions of guardians and children and supports longitudinal assessment of quality of life across general health domains.^17,21^

Some studies found a negative impact of anterior open bite on OHRQoL.^5,8,15^This can be explained by the diagnostic criteria used by these studies, ^24,25^ which did not establish a specific cut-off point for anterior open bite. Increased overbite and increased overjet, on the other hand, are diagnosed when the vertical (overbite) or horizontal (overjet) discrepancy exceeds 2 mm. Therefore, cases are not dichotomized by severity. Severe cases are more likely to be reported by parents, as they are more noticeable, and also because they have a greater potential to negatively affect children’s OHRQoL.^20^

In the present study, posterior crossbite and reduced overbite had a negative impact on children’s self-reported quality of life, while edge-to-edge overjet was associated with worse quality of life in the perception of parents or guardians. Many preschool-aged children maintain non-nutritive sucking habits, such as sucking fingers or pacifiers, leading to aesthetic and functional changes caused by abnormal tooth alignment.^26^ Children with posterior crossbite may have reduced chewing strength and asymmetric function of the masticatory muscles, increasing their susceptibility to signs and symptoms of temporomandibular dysfunction.^27^

Posterior crossbite and reduced overbite had a negative impact on OHRQoL, as reported by preschoolers. These malocclusions can cause functional and aesthetic impairment, but according to the findings observed, posterior crossbite affected quality of life for functional reasons, as reflected in the general and oral health domains of the quality-of-life questionnaire. Reduced overbite can also cause aesthetic concerns. Unfavorable dental and facial aesthetics are considered determinants of social and individual perceptions, playing a key role in the assessment of quality of life.^28^ Malocclusions are associated with bullying and low self-esteem.^4^ In this context, reduced overbite affected not only the oral health domain but also the emotional domain.

Children with edge-to-edge overjet had worse OHRQoL. This type of malocclusion affected the social and physical capacity domains, given that aesthetic impairment facilitates detection by parents and hinders socialization in the school environment. Therefore, some parents feel guilty when their children have this condition.^2,4^

Dental caries, DDE, and history of dental trauma were investigated as possible confounding variables in this study. Furthermore, it has been suggested that the association between oral health and quality of life is influenced by personal and environmental variables. This demonstrates the importance of evaluating demographic factors, such as family income and type of preschool (private or public), which can determine how malocclusions interfere with these children’s daily activities,^8,29^ as demonstrated in the present study.

The findings of this study particularly highlight the relevance of dental caries, which was diagnosed in 50% of the preschool children and negatively impacted OHRQoL. This impact was reported not only by the children’s reports (physical capacity and oral health domains) but also by their parents’ or guardians’ reports (physical capacity, emotional aspect, school activity, oral health, and total score domains). This is because dental caries is a localized condition, as it is commonly associated with the presence of painful symptoms.^6,30^

Some limitations of this study should be acknowledged. Cross-sectional studies are subject to recall bias on the part of participants, as the perceptions of guardians and children may have been influenced by the awareness of the presence or absence of oral changes. Additionally, the findings are context-specific and may not be generalizable to populations with different cultural or socioeconomic backgrounds. A notable strength of this study is its population-based design and the use of validated questionnaires. This study provides relevant insights that can guide oral health professionals, policymakers, and parents/guardians in early identification and management of malocclusions. The assessment of quality of life as a complement to the diagnosis of malocclusions in the primary dentition helps identify demands and treatment needs, prioritize care, establish public health policies, and allocate financial resources for the prevention and management of malocclusions in the permanent dentition. This is necessary to democratize access to dental care in schools, ultimately contributing to improved oral health outcomes among children.^8^

In the present study, children with posterior crossbite were 43 times more likely to report worse OHRQoL. Such a report can represent an excellent indicator of the impacts of this condition and helps guide clinical strategies. This facilitates early intervention by the dentist, leading to less invasive, less painful, and cheaper treatments,^2^ in addition to promoting children’s integration into the school setting, thereby reducing the possible psychosocial damage associated with malocclusions in preschool children.

## Conclusion

To sum up, according to the children’s self-reports, posterior crossbite had a negative impact on the overall quality of life score and oral health domain. Reduced overbite had a negative impact on quality of life in the emotional and oral health domains. In the perception of parents or guardians, edge-to-edge overjet was associated with worse quality of life in the physical capacity and social domains.

## Data Availability

The authors declare that all data generated or analyzed during this study are included in this published article.
